# Intracranial Aneurysm: A Rare Neurological Finding in Ankylosing Spondylitis

**DOI:** 10.7759/cureus.49509

**Published:** 2023-11-27

**Authors:** Mohd Shakirin Pairan, Sanihah Abdul Halim

**Affiliations:** 1 Department of Medicine, Hospital Segamat Ministry of Health, Johor, MYS; 2 Department of Internal Medicine (Neurology), School of Medical Sciences, Universiti Sains Malaysia, Kelantan, MYS; 3 Department of Brain and Behaviour Cluster, School of Medical Sciences, Hospital Universiti Sains Malaysia, Kelantan, MYS

**Keywords:** neurological complications, cervicogenic headache, intracranial aneurysm, hla b27, ankylosing spondylitis

## Abstract

Ankylosing spondylitis can present with various extra-articular manifestations. Vascular complications due to aortic aneurysm or aortitis have been documented. However, an association with intracranial vascular aneurysm is rarely reported. We report a case of a young male with positive HLA B27 ankylosing spondylitis, with extra-articular involvements, presented with recurrent unilateral headache. He was found to have an unruptured anterior communicating artery aneurysm. It was confirmed by a cerebral angiogram, and he was treated conservatively.

## Introduction

Involvement of the nervous system in ankylosing spondylitis (AS) is uncommon and often affects patients with long-standing diseases [1]. The most common neurological complications are spinal cord or nerve root compression due to various mechanisms such as bony impingement, ankylosed spine fracture, atlantoaxial subluxation, or spinal stenosis [1]. Our patient presented with a headache and an intracranial aneurysm, which is rarely reported in AS. Whether this is a part of neuro-vascular complications, or a disease association requires further study.

## Case presentation

A 44-year-old male was diagnosed with ankylosing spondylitis for eight years. The initial symptom was chronic lower back pain with radiological features of sacroiliac joint inflammation. His HLA B27 was positive. He had a strong family history of ankylosing spondylitis and other autoimmune diseases among his first-degree relatives. During the course of his illness, he was found to have extra-articular involvements, which include restrictive lung disease and apical lung fibrosis, as well as sick sinus syndrome in which a cardiac pacemaker was inserted. The disease was stable on sulfasalazine as a disease-modifying therapy.

He had recurrent headaches with neck pain, which was initially treated as a tension-type headache. However, for the past three months, he noticed some changes in the headache character which he described as a persistent, daily, unilateral headache that was only temporarily and partially relieved by analgesic therapy. The headache increased in intensity after he woke up from sleep. It was associated with dizziness, neck pain, nausea and occasionally vomiting especially in the morning. There were no preceding auras, photophobia, or phonophobia to suggest migraine headaches. No diplopia, seizures, or any other focal neurological symptoms. His family history was negative for intracranial hemorrhage or aneurysm. He had stopped smoking for 20 years ago.

On clinical examination, his blood pressure was normal. There were no meningeal signs, papilloedema, or focal neurological deficits. Other systems examination did not indicate any new systemic involvements. There was mild tenderness over the posterior neck region.

**Figure 1:**
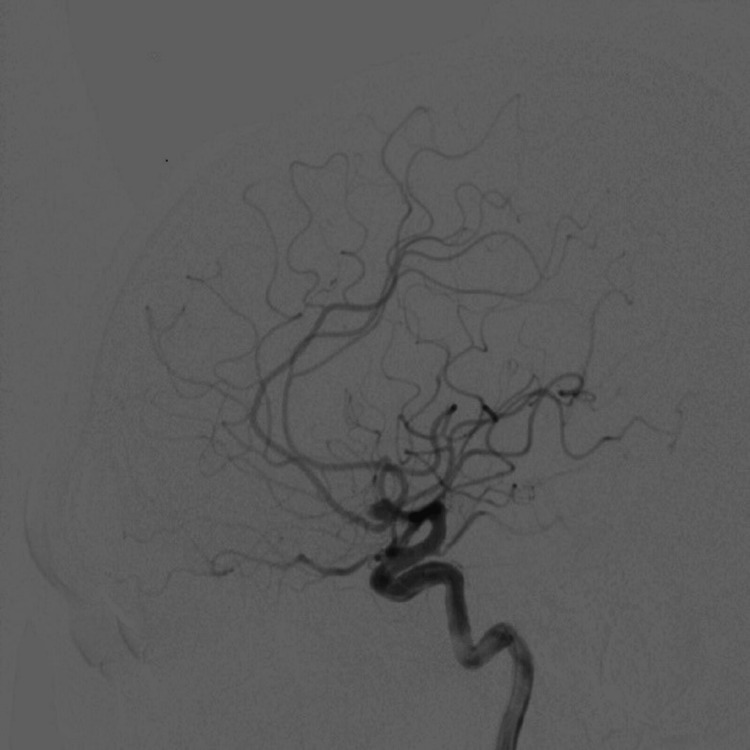
An angiographic image of intracranial artery shows presence of saccular aneurysm at anterior communicating artery.

**Figure 2:**
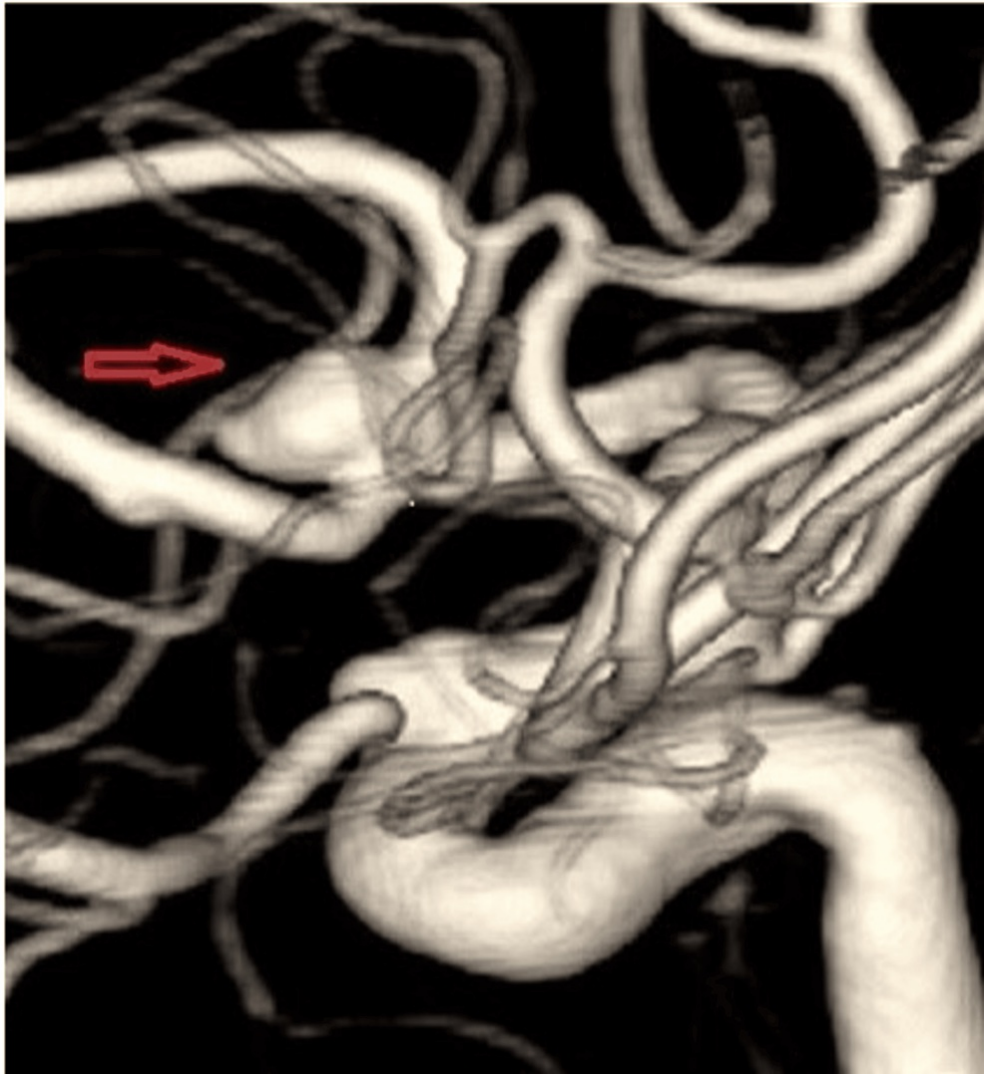
An enlarged angiographic image of the saccular aneurysm (red arrow).

A contrast-enhanced computed tomography (CECT) of the brain was performed and revealed a finding of intracranial aneurysm. This was confirmed by a cerebral angiogram, which showed a saccular aneurysm at the anterior communicating artery (ACom) with a size of 5.7 x 4.0 x 4.0 mm (Figures 1, 2).

A lumbar puncture was performed to look for other secondary causes of headache. The cerebrospinal fluid (CSF) was clear and colorless with normal opening pressure. CSF biochemical tests such as glucose and protein, as well as infectious screening were normal. No features of sinusitis or mastoiditis on head CT. Magnetic resonance imaging (MRI) of the cervical spine showed mild degenerative changes at C4 to C7 spine, but no evidence of spinal canal stenosis or nerve root compression. His electroencephalogram (EEG) was normal. Unremarkable results were obtained from serum lipids, glucose, erythrocyte sedimentation rate, VDRL, and echocardiogram. Other connective tissue disease and vasculitic screening were negative.

He was given analgesic therapy which partially relieved his headache. He was referred to a neurosurgeon and an interventional radiologist for further management of unruptured intracranial aneurysm (UIA). However, in view of the aneurysmal size being small, less than 7mm, it was treated conservatively, with a follow-up MRI after six months. The initial decision to treat conservatively was made in view of the low risk of aneurysmal rupture and there was a possibility of cervicogenic headache. Reoccurrences of headaches during follow-upcoming may warrant a repeat neuroimaging and a reassessment of the risk of rupture. In this case, there was no worsening of headache or new neurological symptoms during subsequent follow-up visits.

## Discussion

Ankylosing spondylitis is a chronic inflammatory joint disease of a young male, that predominantly involves the spine and the sacroiliac joints. It can be associated with various extraarticular manifestations. Bony impingement, ankylosed spine fracture, atlantoaxial subluxation, or spinal stenosis are some of the mechanisms that can lead to neurological complications such as spinal cord compression or nerve root entrapment [1]. These complications may present as axial neck pain or cervicogenic headache [2]. A study of 1,472 Brazilians with AS, found that only 0.9% had neurological manifestations. The most common were atlantoaxial subluxation and cauda equina syndrome [3].

Intracranial aneurysm is rarely reported as a manifestation of AS [4]. However, other vascular complications due to aortic aneurysm dissection or aortitis with aortic valve disease have been documented [5]. The histologic changes in aortic aneurysms support a few mechanisms of its formation; lack of elastic tissue, connective tissue hyalinization, infiltration of lymphocytes and plasma cells, and wall scarring [6,7]. Whether intracranial aneurysms can develop via the same mechanisms was not previously investigated.

Zali et al. reported two cases of AS patients with positive HLA B 27, who presented with life-threatening subarachnoid hemorrhage (SAH). Both patients had the saccular type of aneurysm, involving the posterior communicating artery (PCom) and basilar tip aneurysm respectively [4]. The third case report described an AS patient who presented with severe exophthalmos due to ethmoid mucocele and was found to have an unruptured left anterior communicating aneurysm [8]. So far, no literature reported on the histological changes of intracranial aneurysms in AS patients. Our patient had no other risk factors for the development of intracranial aneurysms such as hypertension, polycystic kidney disease, type IV Ehlers-Danlos syndrome, Marfan syndrome, coarctation of the aorta or bicuspid aortic valve [9].

The headache that occurred in our patient could also be due to a referred pain from the cervical spine. The cervical MRI showed mild degenerative spine disease. Cervicogenic headache is a chronic hemi-cranial pain due to a referred pain from the bony structures or soft tissues of the neck. In the general population, the prevalence of cervicogenic headache is between 0.4% and 2.5%, and the mean age of 42.9 years [10]. The pain in cervicogenic headaches can be produced by active neck movement or passive neck extension with rotation toward the side of the pain. Applying pressure to the involved facet regions or over the ipsilateral greater occipital nerve can trigger the pain. Other causes of unilateral headaches such as migraine, hemicrania continua, or tension-type headaches may have some similar features and may be difficult to distinguish from cervicogenic headaches. Treatment for cervicogenic headache includes analgesic and other neuropathic pain modulators such as tricyclic antidepressants and some antiseizure medications such as carbamazepine, topiramate, and gabapentin as well as physiotherapy [10].

For the treatment decision of an UIA, it should be assessed by a multidisciplinary team. Preventive aneurysm repairs depend on the aneurysm-related risk factors for rupture, such as the size, location, lobulation, previous SAH, age, family history of UIA or SAH, smoking, hypertension, and UIA growth of 1mm or more in any direction [11]. Aneurysm size >12 mm was found to be a significant predictor of poor outcomes [9]. In UIA, most published studies suggest a follow-up imaging of six to 12 months after the initial presentation, followed by every one to two years if the UIA is stable [9]. In our patient, the aneurysm was identified early, thus frequent monitoring and preventive surgical intervention such as aneurysmal clipping can be timely performed if there are signs of impending aneurysmal rupture.

## Conclusions

Cervicogenic headache commonly occurs in ankylosing spondylitis, and it can mimic common primary headaches such as migraines or tension-type headaches. In our case, the size of the aneurysm is small, thus there is also a possibility of a cervicogenic headache rather than an aneurysm-related headache. It is unclear whether intracranial aneurysm in ankylosing spondylitis is an under-reported disease manifestation or just an incidental finding. Further research is required to determine the link between intracranial aneurysm and ankylosing spondylitis.

## References

[1] Sofat N, Malik O, Higgens CS (2006). Neurological involvement in patients with rheumatic disease. QJM.

[2] Biondi DM (2000). Cervicogenic headache: mechanisms, evaluation, and treatment strategies. J Am Osteopath Assoc.

[3] Rodrigues CEM, Vieira WP, Bortoluzzo AB (2012). Low prevalence of renal, cardiac, pulmonary, and neurological extra-articular clinical manifestations in spondyloarthritis: analysis of the Brazilian Registry of Spondyloarthritis. Revista Brasileira de Reumatologia.

[4] Zali A, Shahmohammadi MR, Motiei-Langroudi R (2014). Ankylosing spondylitis associated with intracranial aneurysms: report of 2 cases. Int Clin Neurosci J.

[5] Vendramin I, De Gaspari M, Lechiancole A, Bortolotti U, Livi U (2021). Unexpected aortitis mimicking an ascending aorta intramural hematoma in ankylosing spondylitis. Circ Cardiovasc Imaging.

[6] Somer T, Siltane P (1970). Aneurysm of the descending thoracic aorta, amyloidosis and renal carcinoma in a patient with ankylosing spondylitis. Am J Med.

[7] Takagi H, Mori Y, Umeda Y (2003). Abdominal aortic aneurysm with arteritis in ankylosing spondylitis. J Vasc Surg.

[8] Choi JY, Sohn JT, Sung HJ, Shin IW, Ok SH, Lee HK, Chung YK (2009). Anesthetic management for the endoscopic sinus surgery of a patient with coexisting severe cervical spine ankylosing spondylitis and unruptured cerebral aneurysm: a case report. Korean J Anesthesiol.

[9] Thompson BG, Brown RD Jr, Amin-Hanjani S (2015). Guidelines for the management of patients with unruptured intracranial aneurysms: a guideline for healthcare professionals from the American Heart Association/American Stroke Association. Stroke.

[10] Biondi Biondi, David M (2005). Cervicogenic headache: a review of diagnostic and treatment strategies. J Osteopath Med.

[11] Etminan N, de Sousa DA, Tiseo C (2022). European Stroke Organisation (ESO) guidelines on management of unruptured intracranial aneurysms. Eur Stroke J.

